# Case report: Roxadustat overdose in an anemia patient of chronic kidney disease: insight beyond insignificant consequence

**DOI:** 10.3389/fneph.2024.1413496

**Published:** 2024-08-02

**Authors:** Long-Guang Zhang, Xue-Juan Ma, Xiang-Yang Li

**Affiliations:** Department of Nephrology, The University of Hong Kong-Shenzhen Hospital, Shenzhen, China

**Keywords:** roxadustat, anemia, chronic kidney disease, hypoxia-inducible factor, prolyl hydroxylase inhibitor

## Abstract

A 71-year-old man with a 20-year history of grade 3 hypertension experienced kidney dysfunction 2 years earlier. His serum creatinine (SCr) at the time was 140 μmol/L [with estimated glomerular filtration rate (eGFR) of 43.9 ml/min per 1.73m^2^], for which he received irbesartan since. At initial presentation, the spot urine dipstick protein was 1+, with an albumin-to-creatinine ratio of 230 mg/g (0–30) and normal urine sediments. The SCr was 176 μmol/L (eGFR = 32.8 ml/min per 1.73m^2^). The hemoglobulin (Hb) level decreased from 102 to 96 g/L despite oral ferrous succinate 100 mg twice daily starting 2 months ago. Roxadustat (ROXA) 50 mg (body weight, 70 kg) three times weekly was then prescribed. Unfortunately, the patient mistakenly took the drug at 50 mg three times a day (i.e., 1,050 mg instead of the intended 150 mg per week), which was 3.5 times the recommended starting dose for non-dialysis-dependent chronic kidney disease (CKD) patients (100 mg three times weekly for body weight >60 kg) and two times the highest drug manual-recommended weekly dose (2.5 mg/kg three times weekly) approved in the country. When the attending nephrologist discovered the misuse 1 month later, the patient reported no apparent discomfort, and his home blood pressure was in the range 110–130/60–80 mmHg. Repeat blood tests showed that the Hb increased from 96 to 163 g/L and the SCr from 199 to 201 μmol/L in a month. The serum alanine transaminase (ALT) remained within the normal range (from 12 U/L at baseline to 20 U/L), while the serum total and indirect bilirubin levels were slightly elevated. ROXA was withheld immediately. In 30 days, the serum bilirubin returned to baseline, but the Hb decreased from 163 to 140 g/L, and then to 108 g/L after 3 months. On the other hand, the SCr increased from 179 to 203 μmol/L. At 9 months after the initial dosing, when the SCr increased to 256 μmol/L and the Hb decreased to 94 g/L again, ROXA 50 mg three times weekly was reinitiated uneventfully. Herein, by introducing a case who erroneously consumed twice the highest recommended dose of ROXA for a month, but had apparently no obvious discomfort or unfavorable consequence, we attempt to provide a brief overview of the mechanism of action, characteristics, drug metabolism, and side effect profile associated with this agent.

## Introduction

Anemia is a common complication of chronic kidney disease (CKD) that occurs more frequently with declining renal function. Erythropoietin (EPO) inadequacy, abnormal iron metabolism, systemic inflammation, blunted bone marrow response, and nutritional deficiency, along with a reduced erythrocyte lifetime, are the major causes of anemia of CKD (ACKD) (1, 2). Successful cloning of the *EPO* gene in the early 1980s and the ensuing development of recombinant *EPO* and its analogs enabled erythropoiesis-stimulating agents (ESAs), along with iron supplementation, to become the mainstay of treatment for renal anemia (3). Roxadustat (ROXA) is a first-in-class orally administered hypoxia-inducible factor–prolyl hydroxylase inhibitor (HIF-PHI) that primarily works by promoting the EPO production and iron availability in the treatment of ACKD. It has an established efficacy in an array of phase 3 clinical trials over placebo or ESAs. Notwithstanding, whether ROXA and generics could play a predominant role in ACKD depends on their safety profile, such as the risks of thrombosis and cardiovascular events and the likelihood of oncogenicity (4), which warrant due post-market clinical observation and quality investigations. In this paper, by introducing the case of a patient who erroneously took twofold the highest recommended dose of ROXA for a month, we attempt to provide a brief overview of the mechanism of action, the characteristics, the drug metabolism, and the common side effect profile of this agent.

## Case presentation

**Figure 1:**
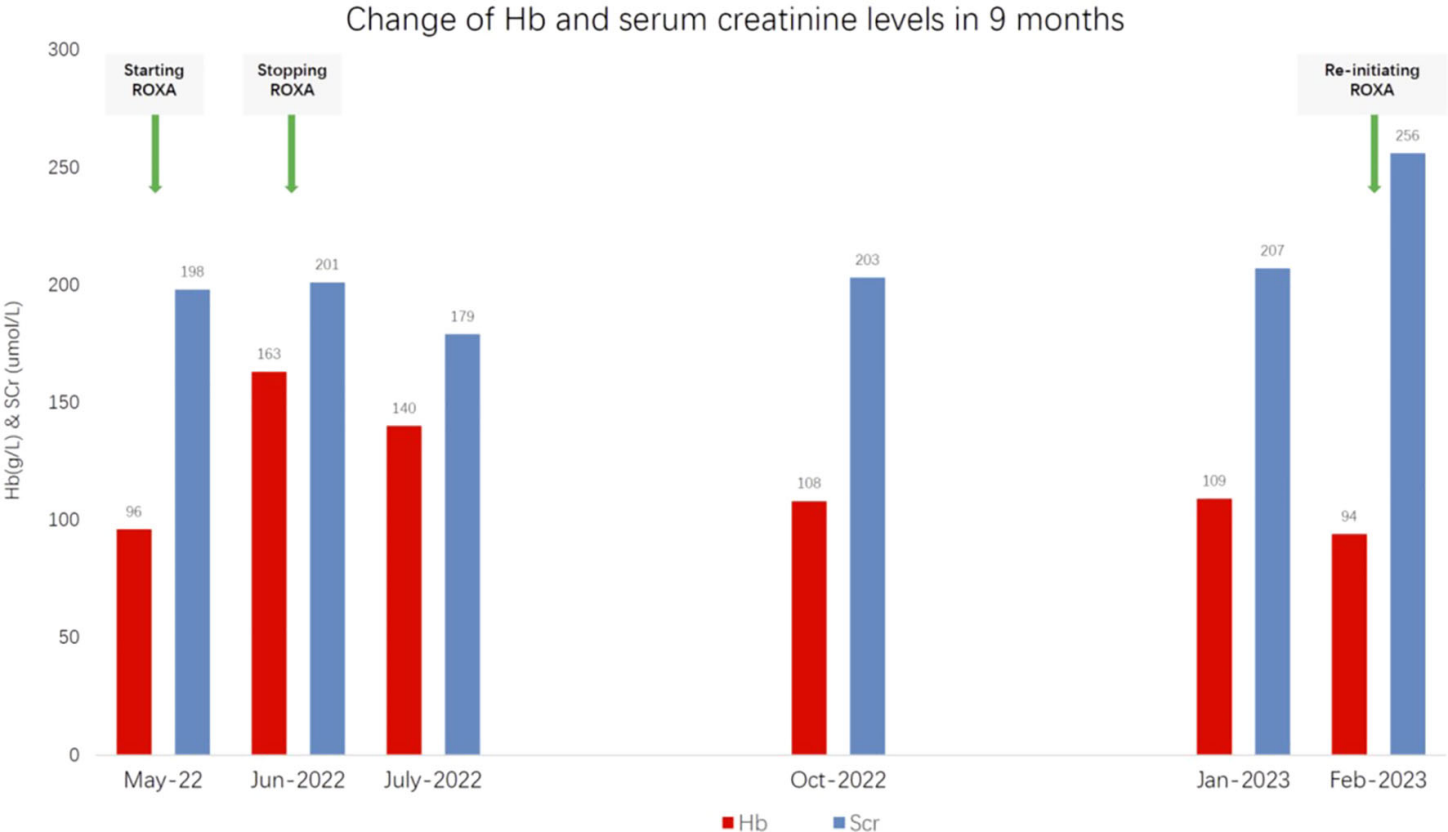
Changes in the hemoglobulin (Hb) and serum creatinine levels 9 months after the initiation of roxadustat (*ROXA*).

A 71-year-old man with a 20-year history of grade 3 hypertension presented for renal dysfunction. He was a retired farmer and non-smoker with otherwise insignificant past medical or family history. His hypertension was sub-optimally controlled at 130–150/90–100 mmHg with 5 mg amlodipine daily. He discovered kidney functional impairment 2 years earlier. His serum creatinine (SCr) at the time was 140 μmol/L [estimated glomerular filtration rate (eGFR) of 43.9 ml/min per 1.73m^2^], for which he received irbesartan, metoprolol extended-release tablet, and atorvastatin. At presentation, the spot urine dipstick protein was 1+, with an albumin-to-creatinine ratio of 230 mg/g (0–30) and normal urine sediments. His SCr decreased from 193 to 176 μmol/L (eGFR = 32.8 ml/min per 1.73 m^2^) after 150 mg irbesartan was switched to sacubitril/valsartan 50 mg plus amlodipine 5 mg daily. The serological examinations for infections, autoimmunity, monoclonal gammopathy, and other potential secondary causes of renal dysfunction were negative. Ultrasonography revealed patent bilateral renal arteries and basically normal kidney sizes. He was diagnosed with hypertensive nephropathy and CKD A2G3b. When low-dose furosemide and spironolactone (20 mg of each agent daily) were taken due to emerging distal leg edema, the home blood pressure was in the range 100–130/54–70 mmHg and his SCr was again elevated from 177 to 199 μmol/L. The diuretics were discontinued. However, the hemoglobulin (Hb) level decreased from 102 to 96 g/L despite oral ferrous succinate 100 mg twice daily starting 2 months prior. Given his old age and the elevated risks of cardiovascular disease, mild anemia, and stage 3 CKD in the absence of chronic comorbidities, as well as in an attempt to avoid a potential quick Hb rise in the short term, ROXA 50 mg three times weekly was prescribed (instead of 100 mg three times a week for a patient with a bodyweight of 70 kg) as a trial treatment. Unfortunately, the patient mistakenly took the drug at 50 mg three times a day (i.e., 1,050 mg instead of the intended 150 mg per week), which was 3.5 times the recommended starting dose for non-dialysis-dependent CKD patients (100 mg three times weekly for body weight >60 kg) and two times the highest drug manual-recommended weekly dose (2.5 mg/kg three times weekly) approved in the country. When the attending nephrologist found the misuse 1 month later, the patient reported no apparent discomfort, and his home blood pressure was in the range 110–130/60–80 mmHg. Repeat blood tests showed that the Hb level increased from 96 to 163 g/L and the SCr from 199 to 201 μmol/L in a month. The serum alanine transaminase (ALT) remained within the normal range (from 12 U/L at baseline to 20 U/L), while the serum total and indirect bilirubin levels increased from 9.6 to 21.7 μmol/L (0–21) and from 4.5 to 12.4 μmol/L (0–5), respectively. The serum creatine kinase was not determined due to the absence of muscular discomfort. ROXA was withheld immediately. After 30 days, the serum bilirubin returned to baseline; however, the Hb decreased from 163 to 140 g/L and subsequently to 108 g/L after 3 months. On the other hand, the SCr increased from 179 to 203 μmol/L. At 9 months after the initial dosing, when the SCr increased to 256 μmol/L and the Hb decreased to 94 g/L again, ROXA 50 mg three times weekly was reinitiated uneventfully. The baseline values and fluctuations of Scr and Hb within 9 months are shown in detail in **Figure 1**.

## Discussion

The essential function of red blood cells (RBCs) is oxygen delivery. ACKD impairs the patient’s health-related quality of life and is associated with increased morbidity and mortality, particularly when Hb falls below 100 g/L. The introduction of epoetin four decades ago revolutionized the treatment for anemia. However, evidence revealed that full anemia correction for a Hb target of above 130 g/L with ESAs is related to the increased incidence of cardiovascular events, hypertension, thrombosis, or stroke. Although the underlying pathophysiology remains so far inadequately elucidated, the proposed mechanisms include, but are not limited to, a supra-physiological concentration of EPO during treatment, extensive presence of the EPO receptor (EPOR) in organ tissues, endothelial activation, and enhanced platelet reactivity (5). In 2012, the KDIGO guideline recommended that oral iron therapy for 1–3 months be applied before the start of ESA treatment until the Hb falls to below 100 g/L, a near-normal correction of anemia to a Hb target of ≤115 g/L, that ESAs not be used to intentionally maintain the Hb concentration above 130 g/L, and that individualization of treatment be applied (3).

HIF is a heterodimeric transcription factor discovered during the search for transcriptional regulators of EPO. It plays a central role in the adaptive response of cells to changes in oxygen availability. HIF consists of an oxygen-labile α-subunit and a constitutive β-subunit. HIF-α has three isoforms. HIF-1α is widely expressed across cell types and is induced more strongly by severe hypoxemia. The expression of HIF-2α is organ-specific (e.g., endothelial cell, kidney, liver, and gut) and is sensitive to moderate hypoxemia. HIF-3α, which possesses multiple variants, supplements or acts as a negative regulator of HIF-1α/HIF-2α. The activity of HIF relies on HIF-α, whose level is tightly regulated by the prolyl hydroxylase domain (PHD). In normoxia, co-factored by Fe^2+^ and using molecular oxygen and 2-oxoglutarate (2-OG) as substrates, PHD hydroxylates two prolyl residues of HIF-α, leading to its ubiquitylation and subsequent proteasomal degradation by the von Hippel–Lindau protein–E3 ligase complex. HIF-α has a mean half-life of only a few minutes. The transcription of EPO is fine-tuned by the HIF-2α/PHD2 axis (6). In hypoxemia, the activity of PHD is suppressed, and an increased level of HIF mediates the upregulation of hundreds of target genes that are implicated in erythropoiesis, iron and energy metabolism, angiogenesis, and cell growth and survival, among others, to defend oxygen homeostasis.

ROXA is the first oral HIF-PHI developed to treat anemia in a more “physiological manner“ by simulating moderate hypoxemia. As of 2023, there have been six HIF-PHIs that obtained approval globally, each possessing a similar core structure working as an Fe^2+^ chelator by reversibly binding to the active site of PHD (7). HIF-PHIs act through multiple effects encompassing the induction of the EPO/EPO receptor, promoting iron absorption and transport and heme production, and can effect serum hepcidin reduction (4). They dose-dependently increase endogenous EPO synthesis, but expose patients to lower supra-physiological EPO concentrations. ROXA is a small-molecule carboxylic acid compound, is easily absorbable, and is eliminated via the urine and feces after metabolism, with a mean half-life of 10–12 h among the CKD population. Moderate hepatic impairment, a reduced eGFR, or dialysis does not impact its pharmacodynamics with clinical relevance. The EPO levels in the general population range from 5 to 20 IU/L. Approximately 1–2 mg/kg of ROXA can induce a median peak endogenous EPO level of 110–350 IU/L, which returns to baseline at 48 h. Therefore, a ROXA dose range above 3–4 mg/kg, corresponding to an EPO concentration of higher than 700–1,000 IU/L, is not otherwise recommended (8). Data have shown that ROXA decreases the serum total and low-density lipoprotein (LDL) cholesterol levels (9). Through the inhibition of CYP2C8 and OATP1B1, 100–200 mg of ROXA can increase the drug exposure (area under the curve, AUC) of gemfibrozil and statins by two- to threefold (10), which could predispose rhabdomyolysis in susceptible patients (11). Sevelamer carbonate, calcium acetate, and ferrous sulfate reduce the AUC of ROXA, and a separate dosing of at least 1 h is recommended. Owing to the promotion of EPO production and the pleiotropic effects of HIF, issues on the drug safety of HIF-PHIs include cardiovascular morbidities and risks of thrombosis and tumorigenicity, among others, particularly at higher doses. ROXA is non-inferior to ESAs concerning MACE (major adverse cardiovascular event) among patients on dialysis; however, it did not meet the non-inferior margin agreed upon with the U.S. Food and Drug Administration (FDA) on non-dialysis patients (4, 12). The relative risk of thrombosis is higher in ROXA than in ESA by 1.35 (7.27 *vs*. 5.37 per 100 patient-year) among dialysis-dependent patients (12), which is thought to be related to the interference of HIF with the coagulation system and the induction of plasminogen activator inhibitor expression.

Healthy adults can generate as much as 2 × 10^11^ of erythrocytes daily, with an average life span of approximately 120 days. The lifetime of RBCs in CKD is reduced with deteriorating kidney function, even by half in stage 5 disease (13). The rapid decline of Hb (−14.1%) within the first 30 days in our case was thought to be associated with neocytolysis. Neocytolysis refers to selective hemolysis or the destruction of newly formed RBCs after sudden EPO withdrawal or rapid reduction (such as in the setting of descent from high altitude to sea level, with a 10%–18% fall of Hb detectable after 8–10 days), forming a tier of erythrocyte regulation during environmental change from hypoxia to normoxia (9), a phenomenon preventable with the administration of low-dose EPO (14). The subsequent Hb decline in this case, aside from EPO insufficiency, could also be related to eryptosis, a programmed cell death of erythrocyte analogous to apoptosis in nucleated cells, which is now considered an important contributor to ACKD. The pathomechanism of eryptosis, although has not been completely elucidated, is linked with the overload of cytosolic Ca^2+^ and the breakdown of cell membrane phospholipid asymmetry and phosphatidylserine externalization resulting from stressful insults (e.g., CKD, malignancies, sepsis, diabetes, heart and hepatic failure, among others) (15). Eryptotic RBCs undergo degradation in the reticulo-endothelial system. In our case, the elevated serum indirect bilirubin level after 1 month of ROXA use was an indicator of hemoglobin breakdown and a heightened RBC turnover rate, which is thought to be associated with the enhanced phagocytosis of erythrocytes due to the decreased anti-deoxidant ability of the newly generated RBCs (16) and eryptosis in the context of CKD. The Scr levels almost did not change after 1 month of ROXA overdose. HIF-PHIs have no proven effect on kidney protection yet; hence, it was difficult to associate the decreased Scr (201–179 μmol/L) in the second month in this connection. Given no additional insult, the subsequent gradual deterioration of renal dysfunction in this case was possibly related to the progression of CKD hypertensive nephropathy.

Hypoxemia represents a feature of tumoral tissue, and the adaptive hypoxemic response of malignant cells influences the cellular growth, metastasis, and therapeutic response. EPOR has been identified in a number of cancer cells, and therapy with ESAs has a known effect of negatively impacting the survival of patients with malignancy. For patients with malignancy, ESAs are now recommended specifically for subjects with chemotherapy-induced anemia whose cancer treatment is not curative in intent (17). However, there is a lack of global guidelines or consensus to inform the management of ACKD in patients with cancer. Intravenous iron therapy offers an alternative to ESAs in this setting, although the relevant protocol and the long-term safety of which are yet to be established. Although current phase 3 trials have not shown an increased tumorigenicity by ROXA, clinicians await the results of a dedicated clinical study in this respect. Moreover, as the HIF-PHIs now in use indiscriminately inhibit PHD1–3 and can increase the levels of HIF-α isoforms, which potentially offer advantages in the hypoxemic adaptation of cancer, we argue for caution in this area. Except for specific malignancies, such as von Hippel-Lindau disease and renal clear cell carcinoma, in which cases the overactivation of HIF is directly related to tumorigenesis, HIF-PHIs should be avoided. The prospects of HIF-PHIs in the therapeutics for ACKD lie in the optimization of treatment precision and individualized drug application for a balanced benefit and risk.

## Data availability statement

The raw data supporting the conclusions of this article will be made available by the authors, without undue reservation.

## Ethics statement

This study was performed in accordance with Helsinki Declaration revised in 2013 and has been approved by the Ethics Committee of the University of Hong Kong-Shenzhen Hospital (approval number: [2023]126). Written informed consent was obtained from the participants or from their relatives for the use of their social and medical data for publication of this case report. Written informed consent was obtained from the individual(s) for the publication of any potentially identifiable images or data included in this article.

## Author contributions

L-GZ: Writing – original draft. X-JM: Writing – original draft. X-YL: Writing – review & editing, Conceptualization, Supervision.

## References

[1] Fishbane S Coyne DW. How I treat renal anemia. Blood. (2020) 136:783–9. doi: 10.1182/blood.2019004330 32556307

[2] Weir MR. Managing anemia across the stages of kidney disease in those hyporesponsive to erythropoiesis-stimulating agents. Am J Nephrol. (2021) 52:450–66. doi: 10.1159/000516901 34280923

[3] Drüeke TB Parfrey PS. Summary of the KDIGO guideline on anemia and comment: reading between the (guide)line(s). Kidney Int. (2012) 82:952–60. doi: 10.1038/ki.2012.270 22854645

[4] Ku E Del Vecchio L Eckardt KU Haase VH Johansen KL Nangaku M. Novel anemia therapies in chronic kidney disease: conclusions from a Kidney Disease: Improving Global Outcomes (KDIGO) Controversies Conference. Kidney Int. (2023) 104:655–80. doi: 10.1016/j.kint.2023.05.009 37236424

[5] Stohlawetz PJ Dzirlo L Hergovich N Lackner E Mensik C Eichler HG. Effects of erythropoietin on platelet reactivity and thrombopoiesis in humans. Blood. (2000) 95:2983–9. doi: 10.1182/blood.V95.9.2983.009k27_2983_2989 10779449

[6] Watts D Gaete D Rodriguez D Hoogewijs D Rauner M Sormendi S. Hypoxia pathway proteins are master regulators of erythropoiesis. Int J Mol Sci. (2020) 21(21):8131. doi: 10.3390/ijms21218131 33143240 PMC7662373

[7] Yeh TL Leissing TM Abboud MI Thinnes CC Atasoylu O Holt-Martyn JP. Molecular and cellular mechanisms of HIF prolyl hydroxylase inhibitors in clinical trials. Chem Sci. (2017) 8:7651–68. doi: 10.1039/C7SC02103H PMC5802278 29435217

[8] Czock D Keller F. Clinical pharmacokinetics and pharmacodynamics of roxadustat. Clin Pharmacokinet. (2022) 61:347–62. doi: 10.1007/s40262-021-01095-x PMC8891203 34905154

[9] Mairbäurl H. Neocytolysis: how to get rid of the extra erythrocytes formed by stress erythropoiesis upon descent from high altitude. Front Physiol. (2018) 9:345. doi: 10.3389/fphys.2018.00345 29674976 PMC5896414

[10] Groenendaal-van de Meent D den Adel M Kerbusch V van Dijk J Shibata T Kato K. Effect of roxadustat on the pharmacokinetics of simvastatin, rosuvastatin, and atorvastatin in healthy subjects: results from 3 phase 1, open-label, 1-sequence, crossover studies. Clin Pharmacol Drug Dev. (2022) 11:486–501. doi: 10.1002/cpdd.1076 35182045 PMC9306950

[11] Yang Q Wang X. A case report of rhabdomyolysis caused by the use of roxadustat in the treatment caused by renal anaemia. Int J Clin Pract. (2021) 75:e14011. doi: 10.1111/ijcp.14011 33411966 PMC8243928

[12] US Food and Drug Administration. Roxadustat for the treatment of anemia due to chronic kidney disease in adult patients not on dialysis and on dialysis. FDA Presentation: Cardiovascular and Renal Drugs Advisory Committee Meeting (2021) (Accessed February 11, 2024).

[13] Li JH Luo JF Jiang Y Ma YJ Ji YQ Zhu GL. Red blood cell lifespan shortening in patients with early-stage chronic kidney disease. Kidney Blood Press Res. (2019) 44:1158–65. doi: 10.1159/000502525 31550724

[14] Rice L Alfrey CP. The negative regulation of red cell mass by neocytolysis: physiologic and pathophysiologic manifestations. Cell Physiol Biochem. (2005) 15:245–50. doi: 10.1159/000087234 16037689

[15] Qadri SM Bissinger R Solh Z Oldenborg PA. Eryptosis in health and disease: A paradigm shift towards understanding the (patho)physiological implications of programmed cell death of erythrocytes. Blood Rev. (2017) 31:349–61. doi: 10.1016/j.blre.2017.06.001 28669393

[16] Song J Yoon D Christensen RD Horvathova M Thiagarajan P Prchal JT. HIF-mediated increased ROS from reduced mitophagy and decreased catalase causes neocytolysis. J Mol Med (Berl). (2015) 93:857–66. doi: 10.1007/s00109-015-1294-y 26017143

[17] Bohlius J Bohlke K Castelli R Djulbegovic B Lustberg MB Martino M. : management of cancer-associated anemia with erythropoiesis-stimulating agents: ASCO/ASH clinical practice guideline update. J Clin Oncol. (2019) 37:1336–51. doi: 10.1200/JCO.18.02142 30969847

